# Iliopsoas tendon insertion footprint with surgical implications in lesser trochanterplasty for treating ischiofemoral impingement: an anatomic study

**DOI:** 10.1093/jhps/hnv060

**Published:** 2015-09-09

**Authors:** Juan Gómez-Hoyos, Ricardo Schröder, Ian J. Palmer, Manoj Reddy, Anthony Khoury, Hal David Martin

**Affiliations:** 1. Hip Preservation Center, Baylor University Medical Center, Dallas, TX, USA; 2. Department of Orthopaedic Surgery, University of Antioquia, Colombia; 3. Texas A&M Health Science Center, College of Medicine at Dallas, TX, USA; 4. Department of Bioengineering, University of Texas at Arlington, TX, USA

## Abstract

The objective of this study was to describe the footprint location of the iliopsoas tendon on the lesser trochanter to clarify the surgical implications of the lesser trochanterplasty for treating ischiofemoral impingement. Ten non-matched, fresh-frozen, cadaveric hemipelvis specimens (average age, 62.4 years; range, 48–84 years; 7 male and 3 female) were included. Registered measures included bony parameters of the lesser trochanter (lesser trochanteric area, distances from the tip to the base in a coordinate system, height and area) and tendinous iliopsoas footprint descriptions (areas and detailed location). The mean height of the lesser trochanter was 13.1 (SD ± 1.8) mm, with female having a smaller lesser trochanter on average (11.3, SD ± 2.0). A double tendinous footprint was found in 7 (70%) specimens. The average area of the single- and double-footprint was 211.2 mm^2^ and 187.9 mm^2^, respectively. An anterior cortical area with no tendinous insertion on the anterior aspect of lesser trochanter was present in all specimens and measured 4.9 mm (SD ± 0.6) on average. The mean ratio between the bald anterior wall and the lesser trochanter height was 38% (SD ± 0.05). The iliopsoas tendon footprint is double (psoas and iliacus) in most cases and is located on the anteromedial tip of the lesser trochanter. A bald anterior wall on the bottom of the lesser trochanter indicates that a partial or total lesser trochanterplasty for increasing the ischiofemoral space without detaching partially or entirely the iliopsoas tendon is improbable.

## INTRODUCTION

The concept of ischiofemoral impingement (IFI) was described by Johnson [1] in 1977. Because of the involvement of the lesser trochanter directly on the narrowing of the ischiofemoral space (IFS), this structure has gained increasing interest. IFI is still poorly understood and the reports about the treatment include open [1–3] or endoscopic decompression [4, 5] of the IFS.

Described techniques for IFS decompression include lesser trochanterplasty, ischioplasty or both [1–5]. Lesser trochanterplasty has been recommended as an effective treatment for symptomatic IFI [4, 5], however the alteration of the psoas tendon insertion after lesser trochanteric resection has not been explored.

Recently, Philippon *et al.* [6] published a comprehensive qualitative anatomic description of the surgically relevant bony and soft tissue anatomy of the proximal femur. The iliopsoas tendon and muscle footprint was calculated and described as an inverted teardrop-shaped insertion occupying the entire posterior surface of the lesser trochanter, extending to the junction of the inferior lesser trochanter with the femoral shaft. However, a clear and detailed quantitative anatomic report of the precise location of the iliopsoas tendon footprint on the lesser trochanter has not been yet described.

The objective of this study was to describe the footprint location of the iliopsoas tendon on the lesser trochanter to clarify the surgical implications of the lesser trochanterplasty for treating IFI.

The hypothesis states the iliopsoas tendon is inserted mostly on the tip of the lesser trochanter and, consequently, it would be partially or totally detached even with limited bony resection. This information would not only assist in open or endoscopic IFI decompression but would also refine the surgical technique in terms of how much lesser trochanter can be removed without affecting the psoas tendon insertion and thus aid in planning a more anatomical decompression, considering the ischioplasty as unique or complimentary procedure in selected cases.

## METHODS

Ten non-matched, fresh frozen, cadaveric hemipelvis specimens were used for this descriptive anatomic study. In accordance with the ethical standards of the responsible committee on human experimentation and with the Helsinki Declaration the cadavers were provided by the University of Texas Southwestern Medical Center (Dallas, USA). The exclusion criteria were evidence of previous surgery and lesser trochanteric deformities affecting the iliopsoas tendinous insertion.

The hemipelvis included the sacrum, ischium, ilium and pubis to the distal femur and all related soft tissues. An extensive posterolateral approach was used to dissect the deep gluteal space beneath the gluteus maximus. The femoral insertion of the hip rotators was taken down and the hip capsule and ligamentum teres were detached to remove the femur from the acetabulum. Particular attention was taken exposing the iliopsoas muscle throughout its entire course from the inner pelvis to its insertion on the lesser trochanter. Either the conjoined tendon or the iliacus and psoas major tendon were identified and traced distally to examine the insertion location on the lesser trochanter. A tenotomy was then performed 4 cm proximal from the femoral insertion. The proximal femurs obtained from each specimen were skeletonized preserving carefully the iliopsoas tendon attachment on the lesser trochanter for further analysis.

**Fig. 1.**
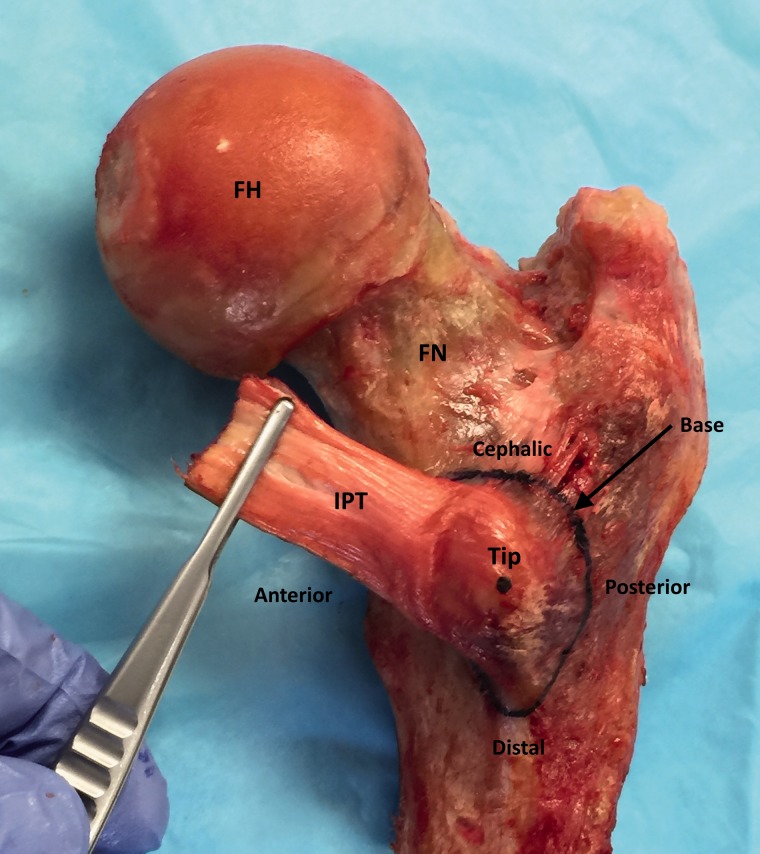
Right hip, posterior view. Lesser trochanter with iliopsoas tendon insertion (single tendon in this case). The tip was marked with a black point and the lesser trochanter base was drawn where the femoral cortex extended beyond the margin of the femoral shaft circumference. The distances between the tip and the anterior, posterior, cephalic and distal base were measured. Observe the insertion of the iliopsoas tendon on the tip and anterior aspect of the lesser trochanter. FN, femoral neck, posterior aspect; FH, femoral head, posterior aspect; IPT, iliopsoas tendon.

A gross anatomic description of the lesser trochanter and iliopsoas insertion was registered for each specimen. Using an anatomic flexible ruler (Covidien, Dublin, Ireland) measurements were recorded in millimeters as follows: (i) Distance from the tip of the lesser trochanter to the anterior base, (ii) Distance from the tip of the lesser trochanter to the posterior base, (iii) Distance from the tip of the lesser trochanter to the cephalic base and (iv) Distance from the tip of the lesser trochanter to the distal base. The tip was marked on the most prominent aspect of the lesser trochanter and the base was drawn where the femoral cortex extended beyond the margin of the femoral shaft circumference (Fig. 1).

**Fig. 2.**
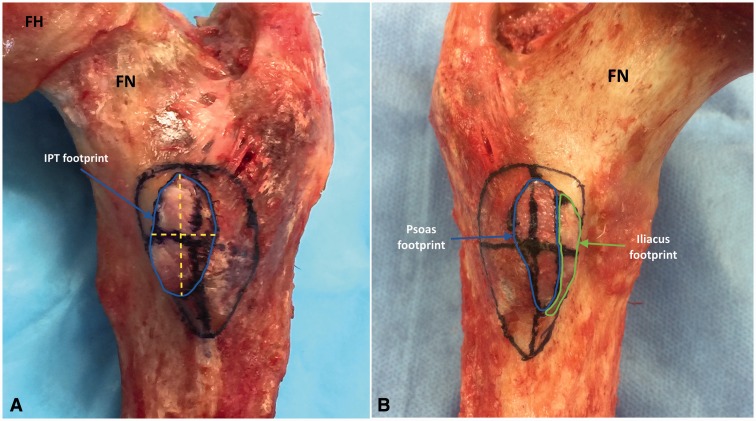
Iliopsoas tendon footprint after tendon resection. A. Right hip, posterior view, single iliopsoas tendon footprint (conjoined). Lesser trochanter after iliopsoas tendon resection. The footprint was drawn with a blue line. The crossing yellow lines represent the coordinate measures for calculating the footprint size. The crossing black lines represent the distances from the tip to the base (coordinates). Observe the ellipse shape of the footprint and the proportion compared with the lesser trochanter size. B. Left hip, posterior view, double footprint. The psoas footprint was drawn in blue color and the iliacus footprint was drawn in green color. FN, femoral neck, posterior aspect; FH, femoral head, posterior aspect.

The iliacus and psoas tendinous insertions were removed sequentially. The borders of the footprint for each tendon were identified and marked with a surgical marker (Viscot Medical, NJ, USA) (Fig. 2A and B). The footprints were measured with a horizontal and vertical line running through the middle aspect of the lesser trochanter (Fig. 2A).

**Fig. 3.**
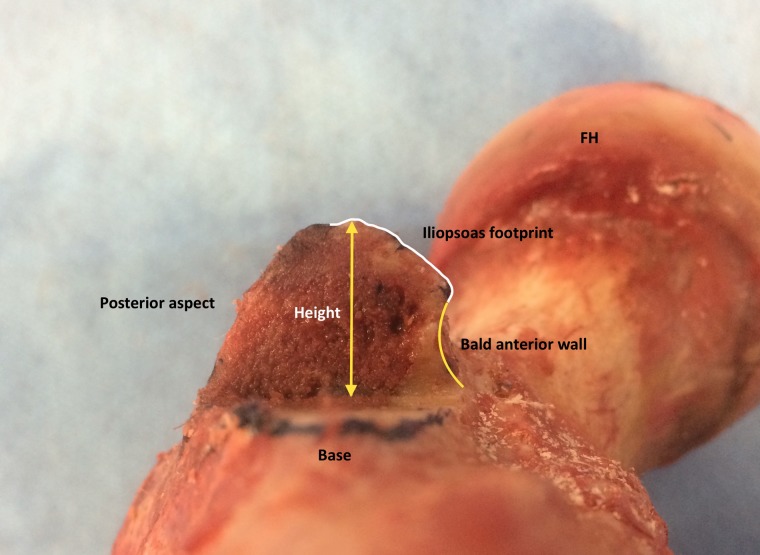
Left hip, view from distal, posterior aspect. The inferior half of the lesser trochanter was removed using a oscillating saw. Observe the height of the lesser trochanter (yellow arrow), the iliopsoas footprint area (white line) and the bald anterior wall (yellow line). The bald anterior wall represents about to 40% of the lesser trochanter height in this case. FH, femoral head, view from distal, posterior aspect.

The distal half of the lesser trochanter was taken down along its axis with a surgical oscillating saw (Stryker, Kalamazoo, USA) and the height of the lesser trochanter and the distance between the anterior base of the lesser trochanter and the footprint border (bald anterior wall) were measured and registered (Fig. 3).

The lesser trochanteric area (defined as the cortical bone surface covering the entire lesser trochanter) and the tendon footprints were calculated by using Microsoft Excel (version 14.0.0, Microsoft, Redmond, WA, USA) with the following equation: Area = π.a.b, where ‘a’ is the major axis and ‘b’ is the minor axis.

Measurements are reported as averages in millimeters and standard deviations. SPSS (version 18.0.0, Chicago, USA) was used for all statistical calculations.

## RESULTS

Ten non-matched, fresh-frozen, cadaveric hemipelvis specimens (average age, 62.4 years; range, 48–84 years; 7 male and 3 female, 5 left and 5 right hips) were finally included in this study and no exclusions were registered.

The shape of the lesser trochanter and the tendinous footprint was elliptical and the tendinous footprint was located on the anteromedial aspect in all cases (Fig. 2A and 2B). Average size of the lesser trochanter in terms of distance from the tip to the base was 17.4 mm (SD ± 2.8) medial, 17.8 (SD ± 3.1) mm lateral, 17.3 (SD ± 3.1) mm proximal, 19.6 (SD ± 4.4) mm distal. The mean height of the lesser trochanter was 13.1 (SD ± 1.8) mm, with female having a smaller lesser trochanter on average (11.3, SD ± 2.0). See Table I.

**Table I.** Lesser trochanter bony parameters with standard deviations

	All specimens (*n* = 10)	Male (*n* = 7)	Female (*n* = 3)
ATBD	17.4 ( ± 2.8)	18.5 (±2.5)	14.6 (±1.1)
PTBD	17.8 (±3.1)	18.7 (±3.3)	15.6 (±1.5)
CTBD	17.3 (±3.1)	18.8 (±1.8)	13.6 (±2.5)
DTBD	19.6 (±4.4)	22.1 (±1.7)	13.6 (±1.5)
LTA	1042.3 (±335.8)	1208.7 (±236.6)	653.9 (±128.3)
LTA/FP Ratio	0.19 (±0.05)	0.18 (±0.05)	0.24 (±0.02)
Bald anterior wall	4.9 (±0.6)	4.9 (±0.5)	5.0 (±1.0)
LTH	13.1 (±1.8)	13.8 (±1.2)	11.3 (±2.0)
Bald anterior wall/LTH ratio	0.38 (±0.05)	0.35 (±0.04)	0.44 (±0.02)

ATBD, anterior tip to base distance; PTBD, posterior tip to base distance; CTBD, cephalic tip to base distance; DTBD, distal tip to base distance; LTA, lesser trochanteric area; FP, footprint ratio; LTH, lesser trochanter height.

**Fig. 4.**
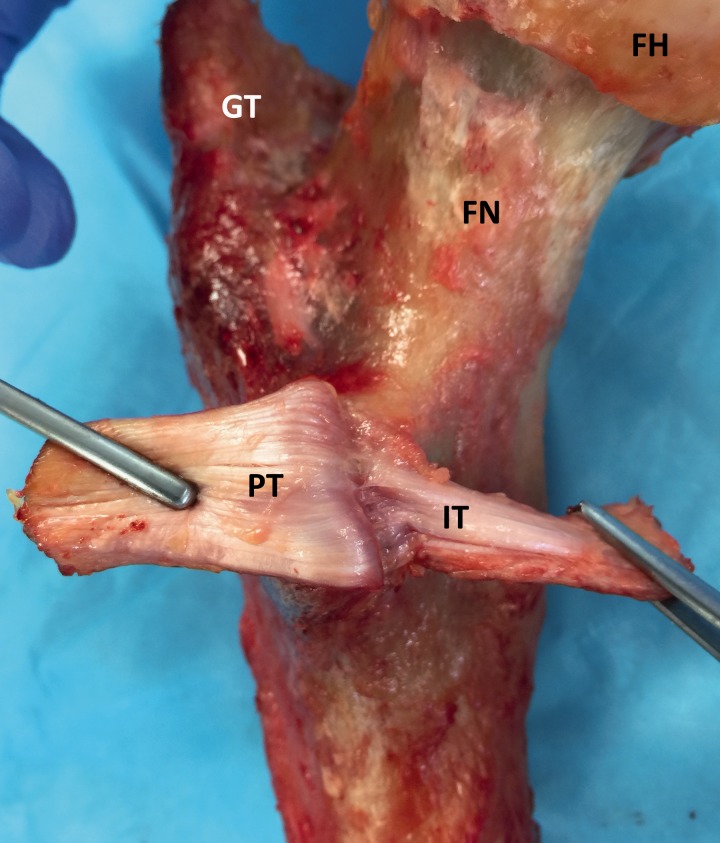
Left hip, posteromedial view. Iliopsoas tendon with independent footprint insertions for the psoas major muscle and iliacus muscle. Observe that the psoas tendon is thicker and its insertion is covering the top of the lesser trochanter, in contrast the iliacus tendon is smaller and its footprint is located on the top of the anterior wall. FH, femoral head; FN, femoral neck; GT, greater trochanter; IT, iliopsoas tendon.

A conjoined tendon, defined as a single footprint for the iliacus and psoas tendon, was found in 3 (30%) specimens and a divided attachment footprints were identified for iliacus muscle and psoas major muscle in 7 (70%) specimens (Fig. 4). When two tendons were identified, the more anterior tendon was always the iliacus and the more posterior (on the tip) was the psoas major tendon. No tendons with more than two heads were identified in this study. The average dimensions of the iliopsoas tendon when inserted as a conjoined structure were 11.6 × 22.6 mm, mean area 211.2 mm^2^. In contrast, the average dimensions of the iliacus and psoas footprint were 5.7 × 18.1 mm, mean area 81.5 mm^2^ and 8.0 × 17.2 mm, mean area 106.4 mm^2^, respectively. When the divided tendons (iliacus and psoas) footprint were added together the mean footprint was 195.0 mm^2^ on average.

The iliopsoas (as a single tendon) and iliacus tendon were noted to be smaller in the female specimens compared with male specimens (Table II).

**Table II.** Mean distances (mm) and footprints (mm^2^) with standard deviations of the psoas and iliacus tendon insertions on the lesser trochanter

	Specimens, *n*(% in subgroup)	Transverse distance, mm (SD)	Cranial-caudal distance, mm (SD)	Mean footprint, mm (SD)
All specimens (*n* = 10)				
Iliopsoas (single insertion)	3	11.6 (±1.52)	22.6 (±4.9)	211.2 (±67.5)
Psoas (independent insertion)	7	8.0 (±1.2)	17.2 (±3.3)	106.4 (±16.6)
Iliacus (independent insertion)	7	5.7 (±1.3)	18.1 (±1.0)	81.5 (±20.5)
Psoas + iliacus footprint	10	–	–	195.0 (±42.3)
Male specimens (*n* = 7)				
Iliopsoas (single insertion)	2	12.5 (±0.7)	25.5 (±0.7)	250.1 (±7.2)
Psoas (independent insertion)	5	8.4 (±1.3)	16.4 (±3.6)	106 (±20.2)
Iliacus (independent insertion)	5	6.2 (±1.3)	18.2 (±1.0)	88.5 (±18.9)
Psoas + iliacus footprint	7	–	–	194.7 (±35.1)
Female specimens (*n* = 3)				
Iliopsoas (single insertion)	1	10.0	17.0	133.5
Psoas (independent insertion)	2	7 (±0.0)	19.5 (±0.7)	107.2 (±3.8)
Iliacus (independent insertion)	2	4.5 (±0.7)	18.0 (±1.4)	64.0 (±14.9)
Psoas + iliacus footprint	3	–	–	171.2 (±11.1)

SD, standard deviation.

**Fig. 5.**
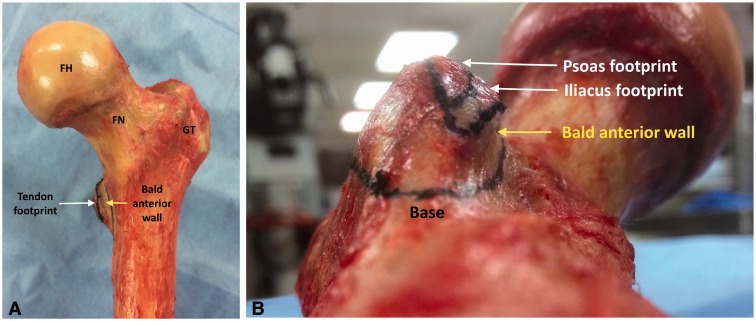
Bald anterior wall. A. Left hip, anterior view, neutral version. Observe the location of the iliopsoas footprint on the top of the lesser trochanter, and the bald wall on the bottom facing anterior. B. Left hip, view from distal, posterior aspect. The bald anterior wall can be observed clearly on this dissection as well as its relationship with the tendinous footprint and lesser trochanteric height. FH, femoral head; FN, femoral neck; GT, greater trochanter.

The mean lesser trochanter area was 1042 mm^2^ on average. This area was noted to be smaller in female (653.9 mm^2^) specimens compared with male (1208.7 mm^2^) specimens. The mean height of the lesser trochanter was 13.1 mm; this measurement was smaller in female (11.3 mm versus 13.8 mm). The footprint area/lesser trochanter ratio was 19% on average (18% in male, 24% in female). An anterior cortical area with no tendinous insertion on the bottom of lesser trochanter was present in all specimens (Figs. 3 and 5). This bald anterior wall was 4.9 mm on average, with no differences between gender. The mean ratio between the bald anterior wall and the lesser trochanter height was 38%; this ratio was noted to be bigger in female (44%) specimens compared with male (35%) specimens.

## DISCUSSION

The iliopsoas footprint on the lesser trochanter is composed of two recognizable areas in the majority of hips (70%) included in this study. The two tendons were mainly inserted on the anteromedial aspect, with the iliacus more anterior than the psoas. A bald anterior wall was found in all specimens (average 38% of the lesser trochanteric height). This bald anterior wall was even greater in females, reaching ∼44% of the lesser trochanteric height. In consequence, the iliacus and psoas tendons inserted on the lesser trochanter would be detached even with a partial lesser trochanterplasty for treating IFI.

The cross-sectional anatomic study of the iliopsoas tendon performed by Blomberg *et al.* [7] included a proportional calculation of the iliopsoas musculotendinous unit at different levels, however, there is no reference about the presence of a separated tendon close to the lesser trochanteric insertion. Tatu *et al.* [8] described a cadaveric study where the psoas tendon was a main tendon with a characteristic rotation arising from the psoas major tendon, an accessory iliacus tendon then progressively fused with the main tendon before it reaches the lesser trochanter.

In spite of the mentioned studies, a recent cadaveric study by Philippon et al, [9] reported the prevalence of a single-, double-, and triple-banded iliopsoas tendon in 28.3, 64.2 and 7.5% of the cadavers, respectively. This study found a similar single- and double-banded proportion. However, no specimens presented a triple-banded insertion in this study.

Regardless of a single- or double-banded tendon, the presence of a bald anterior wall was a consistent finding in all the specimens included in this study. Howse *et al.* [10] in a recent technical note about lesser trochanteric resection for treating symptomatic IFI recommended resecting a few millimeters anterior to the posterior border of the femur to maintain the iliopsoas insertion on the anterior lesser trochanter and femur. Moreover, in the clinical and surgical report by Hatem *et al.* [4] of five confirmed symptomatic IFI cases, they described that an osteoplasty of the posterior one-third of the lesser trochanter was performed to obtain an IFS of at least 17 mm, leaving most of the iliopsoas tendon insertion intact.

Two endoscopic techniques have been described for resecting the lesser trochanter in patients with IFI [10, 11]. However, regardless of the resection technique, to reduce the height of the lesser trochanter could detach the iliopsoas tendon partially or completely according to the results of this anatomic study.

This study described that the iliopsoas tendon insertion is consistently located on the top of the anterior wall, with a bald anterior wall measuring 4.9 mm (SD ± 0.6) on average and representing the 35 and 44% of the lesser trochanteric height in male and female, respectively. The presence of a bald anterior wall in the bottom of the lesser trochanter means that a resection of more than 50% of the tip will detach partially or completely the iliopsoas tendon, being first detached the psoas and then the iliacus tendon according with the location described in this study.

The implications of the resection of the lesser trochanter for treating IFI have not been yet reported. The iliopsoas muscle has been described as an important contributor to hip flexion, trunk flexion and lumbopelvic stabilizer during hip extension and contralateral loading situation [12–15]. The decrease in the force generation due to the surgical procedure could lead in loss of iliopsoas function and consequently to a manifestation of anterior hip pain and low back pain when a psoas pathology is not the indication for releasing it.

Reaching at least 18 mm of IFS is the main purpose when treating IFI [16]. In severe cases even a complete lesser trochanterplasty would not be enough to recover a normal distance as the mean lesser trochanteric height was measured at 13.1 mm (SD ± 1.8).

Truoung *et al.* [2] reported a case of IFI treated successfully with ischioplasty to avoid the detachment of the iliopsoas during a lesser trochanterplasty. Although the implications of ischioplasty for treating IFI have not been yet reported, this is a reasonable treatment in selected cases. For instance, improper positioning of the lesser trochanter because degenerative changes, tumours, previous trauma or surgery could need a concomitant lesser trochanteric resection.

The limitations to this study include the following: first, the specimens consisted of a hemipelvis, which did not allow for correlating anthropometric parameters with the size of the lesser trochanter. Second, the sample included mostly male specimens and all of them were white race, which can affect for the comparative analysis between sexes and races. Third, IFI is sometimes due to neoplastic or traumatic deformities of the lesser trochanter and these findings cannot be extrapolated to those cases. And finally, the footprints and lesser trochanter shape were not a perfect ellipse, which means that the presented footprints and areas are approximations.

## CONCLUSION

The iliopsoas tendon footprint is double in most cases and is located on the anteromedial tip of the lesser trochanter. A bald anterior wall on the bottom of the lesser trochanter averaged 38% of the height indicates that a lesser trochanterplasty for increasing the IFS without detaching partially or totally the iliopsoas tendon is improbable. These findings should warn the surgeons about the partial or complete psoas detachment if the lesser trochanterplasty is the selected method for treating IFI.

## CONFLICT OF INTEREST STATEMENT

None declared.
